# Hypertension prevalence and associated factors among bank workers in Africa, 2024: a systematic review and meta-analysis 

**DOI:** 10.1186/s12889-025-24706-9

**Published:** 2025-10-22

**Authors:** Abdulkerim Hassen Moloro, Abubeker Alebachew Seid, Fikiru Yigezu Jaleta, Abdissa Alemu Dibaba, Eshetu Elfios Endrias, Beminate Lemma Seifu, Bizunesh Fantahun Kase, Kusse Urmale Mare

**Affiliations:** 1 Department of Nursing, College of Medicine and Health Sciences, Samara University, Samara, Ethiopia; 2 Department of Anesthesia, College of Public health and Medical Science, Jimma University, Jimma, Ethiopia; 3 Department of Nursing, School of Nursing, College of Medicine and Health Sciences, Wolaita Sodo University, Wolaita Sodo, Ethiopia; 4 Department of Public Health, College of Medicine and Health Sciences, Samara University, Samara, Ethiopia

**Keywords:** Hypertension, bank workers, systematic review, meta-analysis, Africa

## Abstract

**Background:**

The global burden of hypertension is projected to affect 1.5 billion people by 2025. In Sub-Saharan Africa, the burden currently impacts 74.7 million individuals and is expected to rise sharply to 125.5 million by 2025. However, there is a limited systematic review and meta-analysis that shows pooled prevalence of hypertension among the bank workers population in Africa.. This study aimed to generate updated information on this topic.

**Objective:**

This systematic review and meta-analysis investigated the pooled prevalence of hypertension and associated factors among bank workers in Africa, 2024.

**Methods:**

This systematic review and meta-analysis included cross-sectional studies conducted in African countries and published in English from inception up to December 30, 2024. Excluded were conference proceedings, qualitative research, commentaries, editorial letters, case reports, case series, monthly and annual police reports. The search encompassed full-text publications written in English and databases such as PubMed/MEDLINE, African Journals Online (AJOL), Semantic Scholar, Google Scholar, and Google. Statistical analysis was performed using STATA-17 software and RevMan 5.4. A random-effects model was employed to estimate pooled proportions, and effect sizes with 95% confidence intervals. Funnel plots and Egger’s test were used to examine the possibility of publication bias (p-value < 0.10), and the trim-and-fill method by Duval and Tweedie was applied to adjust for publication bias.

**Results:**

Twelve studies with a total of 3336 study participants that are conducted in four African countries and meet the inclusion criteria were reviewed for this study. Among bank employees in Africa, the overall pooled prevalence of hypertension was 29.75 (95% CI = 23.37, 36.12, I^2^ = 94.4%). Factors such as poor knowledge (OR = 3.55, 95% CI: 2.45,5.14, I^2^ = 0%), family history of hypertension (OR = 4.57, 95% CI: 1.88, 11.12, I^2^ = 75%), physically inactive (OR = 3.81, 95% CI: 2.70, 5.38, I^2^ = 0%), sedentary lifestyle (OR = 2.84, 95% CI: 1.58, 5.12: I^2^ = 0%), and overweight/obesity (OR = 4.01, 95% CI: 2.94, 5.47, I^2^ = 28%) were significantly associated with hypertension.

**Conclusion:**

This study revealed that, approximately one-third of bank employees experience hypertension in Africa. Key modifiable risk factors include poor hypertension awareness, sedentary lifestyles, overweight/obesity, and physical inactivity, along with non-modifiable factors like family history. The findings call for implementing regular health screenings, awareness campaigns about hypertension’s asymptomatic nature, and practical measures like ergonomic adjustments and movement breaks. A multi-sectoral approach combining institutional actions (routine BP monitoring, activity-promoting workplaces) and national policies (health sector prioritization, strengthened primary care) is essential to curb this occupational health crisis effectively. However, this study has several limitations, including significant variability in research findings, a small sample size, reliance on observational studies alone, and the exclusion of non-English publications. Additionally, access to key medical databases such as Scopus, HINARI, EMBASE, CINAHL, and Web of Science was limited.

**Trial registration:**

This study registered in PROSPERO with the registration ID and link as follows: CRD42022364354; https://www.crd.york.ac.uk/PROSPERO/recorddashboard#.

**Supplementary Information:**

The online version contains supplementary material available at 10.1186/s12889-025-24706-9.

## Introduction

Hypertension is the result of persistently high blood pressure in the arteries and specifically described as having two or more readings of ≥ 140 mm Hg for the systolic blood pressure or ≥ 90 mm Hg for the diastolic blood pressure [1]. In the world, it is the primary preventable cause of heart disease, disability (including damage to the heart, kidneys, eyes, brain, and large and peripheral blood arteries resulting from stroke) [2] and mortality [3]. Since 1975, its burden has surged, particularly in low- and middle-income countries, rising from 594 million to 1.13 billion cases by 2015 [4, 5]. Across 182 countries, hypertension rates vary between 13% and 41% [6]., contributing to approximately 7.6 million deaths annually, accounting for 13.5% of global fatalities [7].

Globally, 1.28 billion people aged 30 to 79 have hypertension, with over two-thirds residing in low- and middle-income countries [4, 8]. By 2025, this number is expected to rise to 1.5 billion, with Sub-Saharan Africa experiencing a sharp increase from 74.7 million cases to 125.5 million [9]. Projections indicate that by 2030, 216.8 million Africans will be affected [11–13]. Additionally, the disability-adjusted life-years (DALYs) lost due to hypertension have surged from 95.9 million to 143.0 million, highlighting the growing burden of this condition worldwide [13].

Between 2011 and 2015, cardiovascular disease (CVD) expenses in low- and middle-income countries exceeded USD 3.7 trillion, accounting for nearly 2% of their GDP [14]. Additionally, between 2011 and 2025, non-communicable diseases (NCDs) are projected to result in a total economic loss of $7.28 trillion, equating to an annual loss of $500 billion, or 4% of GDP [14]. Hypertension significantly increases healthcare costs, with affected individuals spending an average of $1,920 more annually, and a total estimated additional expenditure of $131 billion per year [15].

Bank employees including managers, cashiers, clerical staff, and security personnel remain an understudied population in hypertension research, despite frequently experiencing risk factors such as prolonged sitting, mental stress, excessive screen time, and limited physical activity during commutes [16, 17]. Their low-energy occupational lifestyle, typically characterized by physical activity levels of 1.0 to 1.5 metabolic equivalents (METs), is associated with significant health risks, including obesity, diabetes, cardiovascular disease, and depression [19–21]. Moreover, studies indicate that bank workers face an elevated risk of hypertension due to factors such as advancing age [21], excessive salt and saturated fat consumption [21], obesity (BMI ≥ 25) [23–25], and ignorance of the information regarding hypertension, all of which contribute to the higher prevalence of hypertension in this occupational group [17].

By 2025, hypertension is expected to affect three out of four individuals in low- and middle-income nations [25, 26]. To address this growing issue, the Pan-African Society of Cardiology (PASCAR) has proposed ten strategic recommendations to help African health ministers achieve a 25% reduction in hypertension cases by 2025 [27, 28].

Hypertension burden in African nations among bank workers vary significantly, ranging from 12.4 to 52.4%, highlighting the need for further investigation and consistent result [17, 30–40]. Although numerous epidemiological studies exist, no systematic review or meta-analysis has yet synthesized current evidence on hypertension prevalence and associated risk factors specifically among African bank employees, highlighting a critical gap in comprehensive research. The study identifies key research gaps while generating actionable evidence to inform health ministries, policymakers, and community health programs. Importantly, it underscores the necessity for immediate preventive strategies to combat escalating hypertension rates among Africa’s banking workforce.

## Objectives and review questions

This investigation aimed to determine the cumulative level of hypertension and synthesize data on the contributing factors to hypertension among bank workers in Africa. The following review questions provide a framework for this systematic review and meta-analysis: (1) What is the overall prevalence of hypertension among bank workers in Africa? And (2) Which factors contribute to hypertension in this population?

## Methods

### Study report and registration

This systematic review and meta-analysis adhered to the guidelines outlined by the Preferred Reporting Items for Systematic Reviews and Meta-Analyses (PRISMA) statement [40]. The study results were reported following the PRISMA-2020 standard [40, 41]. Additionally, the study protocol was registered with the International Prospective Register of Systematic Reviews (PROSPERO) under the registration identification number CRD42022364354.

### CoCoP and PEO search guide

**Condition**: Hypertension

**Context**: Africa (African bank sector)

**Population**: African bank employees (managers, cashiers, clerical staff, and security personnel)

**Exposure**: The factors or exposures that raise the risk of hypertension

**Outcome**: Hypertension prevalence and its associated factors

### Search strategy and sources of information

Two independent authors (AHM and KUM) designed and executed the search strategy. Published research on the hypertension prevalence and associated factors among bank workers used in Africa was used in the review. A systematic search was conducted for studies published from inception up to December 30, 2024 across multiple electronic databases. The search yielded the following results: Google Scholar (375 studies), African Journals Online (AJOL) (750 studies), PubMed/MEDLINE (2475 studies), and Google/citation searches (15 studies).

All relevant records were included for further screening. As a result of the comprehensive search and further screening, only studies published between 2015 and 2024 were included in the review. For PubMed advanced searching, keywords, free text search terms, and Medical Subject Headings (Mesh) were all used (See Table 1). Bank employees, Africa, hypertension, and related factors were used as stand-in terms, which were then combined using Boolean operators as search phrases. *[“Hypertension” OR” “High blood pressure” OR “Bank employees” OR “Occupational groups “OR” Employees” OR “Associated factors” OR” Risk factors” OR “Africa”]/[“Hypertension” AND” “High blood pressure” AND “Bank employees” AND “Occupational groups “AND” Employees” AND “Associated factors” AND” Risk factors” AND “Africa “AND “African bank sector”]* were utilized as substitute terms and merged using Boolean operators as search phrases. The same search methodology, incorporating these terms, was applied to Google Scholar and AJOL.

To ensure a thorough and comprehensive study, we employed a snowballing technique, examining references from identified publications and mining citations from previous narrative and systematic reviews to uncover additional relevant studies. Additionally, to ensure a thorough search and minimize publication bias, efforts were made to identify unpublished or non-commercially published research, including thesis, dissertations, government reports, and clinical trial registries. Furthermore, we sought recommendations from specialists, experienced librarian, researchers, and relevant organizations to identify another pertinent research that had already been conducted. The search results from Google Scholar, African Journals Online (AJOL), and PubMed/MEDLINE were imported into the reference management software (EndNote**™**) where duplicate entries were removed to ensure efficiency and avoid redundancy.

**Table 1:** PubMed search strategy for the systematic review and meta-analysis of hypertension prevalence and associated factors among bank workers in Africa, 2024

Search Number	Search Detail	Results
*#1*	“Hypertension**”** [MeSH Terms]	***333***,***430***
*#2*	((((Hypertension [Title/Abstract]) OR (Blood Pressure, High [Title/Abstract])) OR (Blood Pressure, High [Title/Abstract])) OR (High Blood Pressure [Title/Abstract])) OR (High Blood Pressures [Title/Abstract]) OR (systolic blood pressure [Title/Abstract]) OR (diastolic blood pressure [Title/Abstract]) OR (systolic hypertension [Title/Abstract]) OR (diastolic hypertension [Title/Abstract])	***583***,***354***
*#3*	“Risk Factors” [MeSH Terms]	***1***,***049***,***217***
*#4*	((((((((((((((((((Risk Factors[Title/Abstract]) OR (Factor, Risk[Title/Abstract])) OR (Risk Factor[Title/Abstract])) OR (Associated factors[Title/Abstract])) OR (Determinant factors[Title/Abstract])) OR (contributing factors[Title/Abstract])) OR (Social Risk Factors[Title/Abstract])) OR (Personal Factors[Title/Abstract])) OR (Environmental Factors[Title/Abstract])) OR (Factor, Social Risk[Title/Abstract])) OR (Factors, Social Risk[Title/Abstract])) OR (Risk Factor, Social[Title/Abstract])) OR (Risk Factors, Social[Title/Abstract])) OR (Social Risk Factor[Title/Abstract])) OR (Health Correlates[Title/Abstract])) OR (Correlates, Health[Title/Abstract])) OR (Population at Risk[Title/Abstract])) OR (Populations at Risk[Title/Abstract])) OR (Risk Scores[Title/Abstract])) OR (Risk Score[Title/Abstract])) OR (Score, Risk[Title/Abstract])) OR (Risk Factor Scores[Title/Abstract])) OR (Risk Factor Score[Title/Abstract])) OR (Score, Risk Factor[Title/Abstract]	***1***,***076***,***795***
*#5*	“Occupational groups“[MeSH Terms]	***780***,***571***
*#6*	((((((((Occupational Groups [Title/Abstract]) OR (Employees [Title/Abstract])) OR (Personnel [Title/Abstract])) OR (Workers [Title/Abstract])) OR (Group, Occupational [Title/Abstract])) OR (Groups, Occupational [Title/Abstract])) OR (Occupational Group [Title/Abstract])) OR (Employee [Title/Abstract])) OR (Organizational [Title/Abstract])) OR (Governmental [Title/Abstract])) (Worker[Title/Abstract])	***27***,***021***
*#7*	((((((Africa [MeSH Terms]) OR (Africa South of the Sahara [MeSH Terms])) OR (Africa, Western [MeSH Terms])) OR (Africa, Southern [MeSH Terms])) OR (Africa, Northern [MeSH Terms])) OR (Africa, Eastern [MeSH Terms])) OR (Africa, Central [MeSH Terms])	***357***,***740***
*#8*	(((((((((((((((((((((((((((((((((((((((((((((((((((((((((((((((((((((((((Africa[Title/Abstract]) OR (Africa South of the Sahara[Title/Abstract])) OR (Sub-Saharan Africa[Title/Abstract])) OR (Subsaharan Africa[Title/Abstract])) OR (Africa, Sub-Saharan[Title/Abstract])) OR (Africa, Western[Title/Abstract])) OR (Africa, West[Title/Abstract])) OR (West Africa[Title/Abstract])) OR (Western Africa[Title/Abstract])) OR (Benin[Title/Abstract])) OR (Burkina Faso[Title/Abstract])) OR (Cabo Verde[Title/Abstract])) OR (Cote d’Ivoire[Title/Abstract])) OR (Gambia[Title/Abstract])) OR (Ghana[Title/Abstract])) OR (Guinea[Title/Abstract])) OR (Guinea-Bissau[Title/Abstract])) OR (Liberia[Title/Abstract])) OR (Mali[Title/Abstract])) OR (Mauritania[Title/Abstract])) OR (Niger[Title/Abstract])) OR (Nigeria[Title/Abstract])) OR (Senegal[Title/Abstract])) OR (Sierra Leone[Title/Abstract])) OR (Togo[Title/Abstract])) OR (Africa, Southern[Title/Abstract])) OR (Southern Africa[Title/Abstract])) OR (Angola[Title/Abstract])) OR (Botswana[Title/Abstract])) OR (Eswatini[Title/Abstract])) OR (Lesotho[Title/Abstract])) OR (Malawi[Title/Abstract])) OR (Mozambique[Title/Abstract])) OR (Namibia[Title/Abstract])) OR (South Africa[Title/Abstract])) OR (Zambia[Title/Abstract])) OR (Zimbabwe[Title/Abstract])) OR (Africa, Northern[Title/Abstract])) OR (Northern Africa[Title/Abstract])) OR (North Africa[Title/Abstract])) OR (Maghreb[Title/Abstract])) OR (Maghrib[Title/Abstract])) OR (Sahara[Title/Abstract])) OR (Algeria[Title/Abstract])) OR (Egypt[Title/Abstract])) OR (Libya[Title/Abstract])) OR (Morocco[Title/Abstract])) OR (Tunisia[Title/Abstract])) OR (Africa, Eastern[Title/Abstract])) OR (East Africa[Title/Abstract])) OR (East Africa[Title/Abstract])) OR (Eastern Africa[Title/Abstract])) OR (British Indian Ocean Territory[Title/Abstract])) OR (Burundi[Title/Abstract])) OR (Djibouti[Title/Abstract])) OR (Eritrea[Title/Abstract])) OR (Ethiopia[Title/Abstract])) OR (Kenya[Title/Abstract])) OR (Rwanda[Title/Abstract])) OR (Somalia[Title/Abstract])) OR (South Sudan[Title/Abstract])) OR (Sudan[Title/Abstract])) OR (Tanzania[Title/Abstract])) OR (Uganda[Title/Abstract])) OR (Africa, Central[Title/Abstract])) OR (Central Africa[Title/Abstract])) OR (Cameroon[Title/Abstract])) OR (Central African Republic[Title/Abstract])) OR (Chad[Title/Abstract])) OR (Congo[Title/Abstract])) OR (Democratic Republic of the Congo[Title/Abstract])) OR (Equatorial Guinea[Title/Abstract])) OR (Gabon[Title/Abstract])) OR (Sao Tome[Title/Abstract] AND Principe[Title/Abstract])	***548***,***457***
*#9*	**#1 OR #2**	***666***,***737***
*#10*	**#3 OR #4**	***1***,***704***,***105***
*#11*	**#5 OR #6**	***29***,***803***
*#12*	**#7 OR #8**	***642***,***031***
*#13*	**#9 AND #10 AND #11 AND #12**	***2475***

### Inclusion criteria

This review included studies employing cross-sectional designs. Although our search was limited to English-language publications, we considered both peer-reviewed journal articles and unpublished reports. We incorporated data from national surveys and country-level studies, with a particular focus on research conducted in Africa. To ensure comprehensive coverage, we included all relevant publications from the earliest available records to the present that reported on hypertension prevalence and associated factors among bank employees. Consequently, the review included studies published from 2015 to 2024.

### Exclusion criteria

Investigations into the pooled prevalence of hypertension among African bank workers did not include studies that do not provide information on the prevalence of hypertension and associated factors, or for which it is not possible to obtain the necessary information even after contacting the authors. The exclusion list included the following: conference proceedings, qualitative studies, commentary, editorial letters, case reports, case series, monthly and annual police reports, and articles published in languages other than English. Furthermore, studies involving pregnant women, individuals with mental health disorders, or chronic conditions such as renal disease and diabetes were excluded to reduce potential confounding factors.

### Definition of outcome

The primary outcome of this study was to determine the pooled prevalence of hypertension and identify associated factors among bank workers in Africa. Hypertension was defined as having two or more readings of ≥ 140 mm Hg for the systolic blood pressure or ≥ 90 mm Hg for the diastolic blood pressure or taking antihypertensive medication [1]. Secondary outcomes included the evaluation of hypertension risk factors, such as demographic factors (age, sex, residence, educational status), family history of hypertension, comorbidities (diabetes mellitus, BMI, central obesity), and lifestyle factors (alcohol consumption, physical inactivity, cigarette smoking, salt intake, khat chewing).

### Selection of studies

After evaluating the studies in accordance with the inclusion and exclusion criteria, two authors (AAS&BFK) selected which ones to include. The review was carried out with reference to the following methodologies. First, the search yielded article titles, which were evaluated. Second, their eligibility was abstractly screened using the predetermined inclusion and exclusion criteria. Lastly, the abstracts of these chosen titles were included in the last round of full-text screening. Using Microsoft Excel**™**, the data charting and screening process was finished. Only studies that both authors approved were included in the full review of the articles. Any disagreements among the authors were settled through discussion or consultation with a third reviewer (EEE). After every article was removed, the final article list for data extraction was produced.

### Data extraction and management

Once every eligible article had been found, the pertinent data was extracted onto a Microsoft Excel spreadsheet by two independent reviewers (FYJ&AHM). The Joanna Briggs Institute (JBI) data extraction form for systematic reviews and research syntheses served as the model for the development of a data extraction format [42]. All review team members participated in an independent test of the data extraction procedure using Microsoft Excel prior to the actual data extraction.

The data extraction tool contained the following information for each included article: the last name of the first author, the year the study was published, the country or region the study was conducted in, the study design, the study period, sample size, response rate, population, proportion of hypertension, related factors, effect size of risk factors, and blood pressure readings used. Throughout the extraction process, disagreements between data extractors were resolved in order to reach a consensus. A third reviewer (BLS) was consulted with the authors in case a consensus could not be reached.

### Quality assessment

The listed studies were assessed independently by two reviewers (AHM&AAS). For prevalence or proportion studies, the JBI checklists [43] were used to assess the articles’ quality. The tool has nine parameters: [1] a suitable sampling frame; [2] a suitable sampling technique; [3] a suitable sample size; [4] a description of the subject and setting of the study; [5] a suitable data analysis; [6] the application of valid methods for the conditions that have been identified; [7] valid measurement for each participant; [8] the application of suitable statistical analysis; and 9) an adequate response rate.

Alternatives such as yes, no, not applicable, and unknown is available in the tools. Responses that were yes received a score of one, while those that were unclear, irrelevant, or absent received a zero. A study was ultimately deemed high quality and included in the final analysis if it received a score of seven or higher. Throughout the critical evaluation, one author (KUM) settled the arguments between the other two.

### Statistical analysis

The data were displayed using tables and graphs according to the findings of the selected study’s conclusions. The STATA 17 version software is used for data entry and analysis because of its adaptation to the Metan program. The random effect model was used to show the pooled prevalence of hypertension among bank employees [44]. We used the Freeman Tuckey variant of the arcsine square root transformation of proportions to avoid variance variability because the random-effects model takes into account sources of between-study variance [45, 46].

Using the I^2^ statistic and a chi-squared test in accordance with Cochran’s Q statistic with a 5% significance level, heterogeneity was measured based on statistical findings, outcome presentations, and methodological [47]. I^2^ values of 25%, 50%, and 75% were considered indicative of low, moderate, and high heterogeneity, respectively [48]. When I^2^ > 50% and p-value less than 0.05, the existence of heterogeneity were declared [48]. Subgroup analyses and meta-regressions were performed to investigate sources of heterogeneity [49]. Further, to ascertain the effect of individual studies on pooled estimates, a sensitivity analysis was also carried out [49].

The RevMan 5.4 version software was utilized to calculate the effect size in order to determine whether there is a significant association between the related factors and hypertension among bank workers. P-values less than 0.05 are used to determine the statistical significance level for effect size.

### Publication bias

For the purpose of examining the possibility of publication bias and small-study effects, funnel plots and Egger’s test [50] were utilized. Publication bias was identified when the p-value was statistically significant (p value < 0.05). A minimum of 10 studies is required to reliably assess publication bias. (51).Based on these criteria, no evidence of publication or small-study bias was found.

## Results

### Study selection and identification

**Fig. 1:**
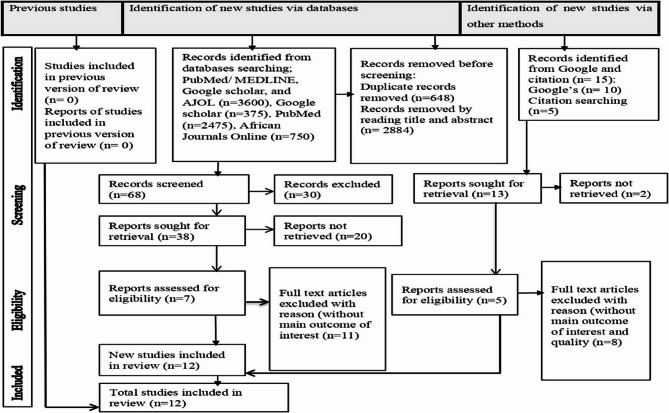
PRISMA (Preferred Reporting Items for Systematic Reviews and Meta-Analyses) flowchart illustrating the study selection process for hypertension prevalence and associated factors among bank workers in Africa, 2024

Initially, 3,600 records were identified across databases including PubMed/MEDLINE (*n* = 2,475), Google Scholar (*n* = 375), and African Journals Online (AJOL) (*n* = 750), with 648 duplicates removed and 2,884 excluded after title and abstract screening. An additional manual search via Google and citation tracking yielded 15 more articles (Google: *n* = 10; citation searching: *n* = 5). From the 68 records screened, 30 were excluded, and 38 full-text articles were sought for retrieval. From the pool of 51 potentially eligible and retrieved studies, 39 were excluded for reasons such as unavailability of full text, inadequate quality, studies not conducted in Africa, or lack of the primary outcome of interest. Ultimately, 12 [17, 30–40] studies met all inclusion criteria and were included in the final review and meta-analysis. The PRISMA flow diagram (See Fig. 1) outlines the screening procedure in detail.

### Assessment of methodological quality for included studies

 This review included 12 studies, with methodological quality ranging from moderate to high. According to the JBI critical appraisal checklist [43], which was used for studies that reported prevalence data, six studies [17, 29, 31, 34, 36, 38] scored 9 points, three studies [30, 32, 33] scored 8 points, and the remaining 3 studies [35, 37, 39] scored 7 points (Table 2).

**Table 2:** Methodological quality of included studies on hypertension prevalence and associated factors among bank workers in Africa, 2024

Authors and year of publication	Q1	Q2	Q3	Q4	Q5	Q6	Q7	Q8	Q9	Quality score/9
Dejenie et al. (2021)[31]	✓	✓	✓	✓	✓	✓	✓	✓	✓	9
Fikadu G et al. (2017)[33]	U	✓	✓	✓	✓	✓	✓	✓	✓	8
Zewde GT et al. (2020)[34]	✓	✓	✓	✓	✓	✓	✓	✓	✓	9
Kassie A et al. (2021)[38]	✓	✓	✓	✓	✓	✓	✓	✓	✓	9
Hinson AV et al. (2019)[39]	U	U	✓	✓	✓	✓	✓	✓	✓	7
Hailu Dagne et al. (2021)[31]	✓	✓	✓	✓	✓	✓	✓	✓	✓	9
Ebhohimen KC et al. (2017)[30]	✓	✓	✓	✓	✓	✓	✓	✓	U	8
Diwe KC et al. (2015)[32]	✓	✓	✓	✓	✓	✓	✓	✓	U	8
Sawsan A et al. (2022)[37]	U	U	✓	✓	✓	✓	✓	✓	✓	7
Khaild et al. (2022)[35]	U	U	✓	✓	✓	✓	✓	✓	✓	7
Yirgalem Amare et al[29]	✓	✓	✓	✓	✓	✓	✓	✓	✓	9
Ojo OY et al. (2024)[36]	✓	✓	✓	✓	✓	✓	✓	✓	✓	9

✓ = yes, X = no, U = unclear

### Characteristics of included studies

This systematic review and meta-analysis included 12 original publications that were conducted in four African nations. Among these, six (50%) were conducted in East African nations (Ethiopia) [17, 29, 31, 33, 34, 38], four (33.33%) in West African countries (3 from Nigeria) [30, 32, 36] and (1 from Benin) [39], and 2 (16.66%) studies were in North African countries (from Sudan) [35, 37]. Among the included studies, 8 (66.67%) studies published after 2019. All the included studies were cross-sectional surveys and institutional-based (Bank sector). A total of 3336 participants were included in this systematic review and meta-analysis. Additionally, the review included a total of 2,027 male and 1,309 female participants, resulting in an overall male-to-female ratio of 1.55:1(Table 3).

**Table 3:** Characteristics of included studies for hypertension prevalence and associated factors among bank workers in Africa, 2024

Authors and year of publication	Sub-region	Country	Study year	Study setting	Study design	Criteria for HTN Diagnosis	Sample size	Sex ratio (M: F)	Prevalence of HTN
Dejenie et al. (2021)[17]	East African	Ethiopia	2020	Facility	Cross sectional	≥ 130/80 mm/Hg	513	394:119 (3.31:1)	127(24.8%)
Fikadu G et al. (2017)[33]	East African	Ethiopia	2010	Facility	Cross sectional	≥ 140/90 mmHg	554	333:221 (1.51:1)	106(19.13%)
Zewde GT et al. (2020)[34]	East African	Ethiopia	2018	Facility	Cross sectional	≥ 140/90 mmHg	149	90:59 (1.53:1)	41(27.5%)
Kassie A et al. (2021)[38]	East African	Ethiopia	2020	Facility	Cross sectional	≥ 130/80 mm/Hg	368	285:83 (3.43:1)	193(52.4%)
Hinson AV et al. (2019)[39]	West African	Benin	2016	Facility	Cross sectional	≥ 140/90 mmHg	225	117:108 (1.08:1)	57(25.3%)
Hailu Dagne et al. (2021)[31]	East African	Ethiopia	2017	Facility	Cross sectional	≥ 140/90 mmHg	149	90:59 (1.53:1)	41(27.5%)
Ebhohimen KC et al. (2017)[30]	West African	Nigeria	2016	Facility	Cross sectional	≥ 140/90 mmHg	400	193:207 (0.93:1)	149(37.3%)
Diwe KC et al. (2015)[32]	West African	Nigeria	2015	Facility	Cross sectional	≥ 140/90 mmHg	194	100:94 (1.06:1)	24(12.4%)
Sawsan A et al. (2022)[37]	North African	Sudan	2022	Facility	Cross sectional	≥ 140/90 mmHg	187	112:75 (1.49:1)	55(29.4%)
Khaild et al. (2022)[35]	North African	Sudan	2020	Facility	Cross sectional	≥ 130/90 mmHg	98	72:26 (2.77:1)	45(45.9%)
Yirgalem Amare et al(2022)[29]	East African	Ethiopia	2021	Facility	Cross sectional	≥ 140/90 mmHg	253	129:124 (1.04:1)	59(23.3%)
Ojo OY et al. (2024)[36]	West African	Nigeria	2021	Facility	Cross sectional	≥ 140/90 mmHg	246	112:134 (0.84:1)	82(33.33%)

### Pooled prevalence of hypertension among bank workers in Africa

**Fig. 2:**
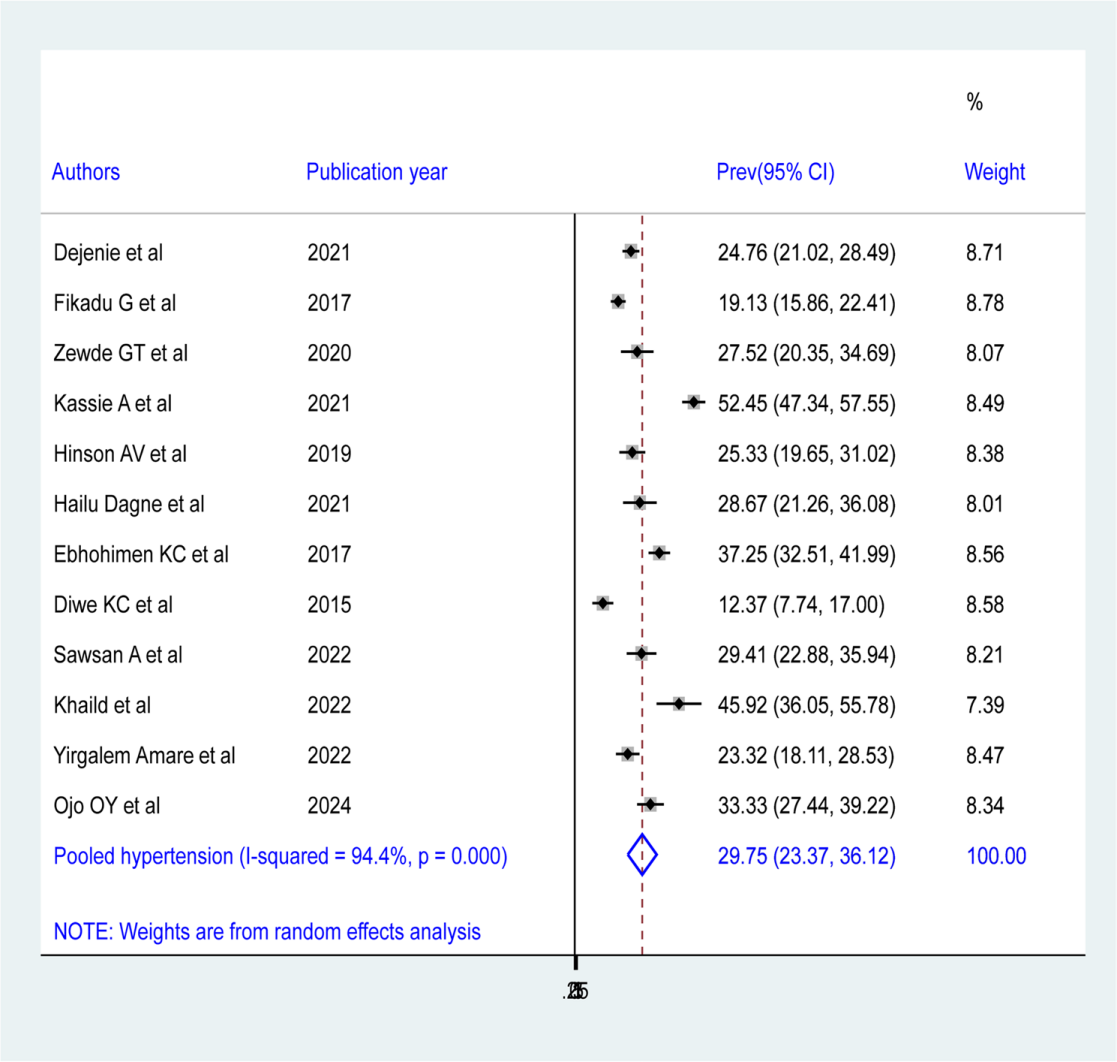
Forest plot showing the pooled prevalence of hypertension among bank workers in Africa from random-effect model analysis, 2024

**Fig. 3:**
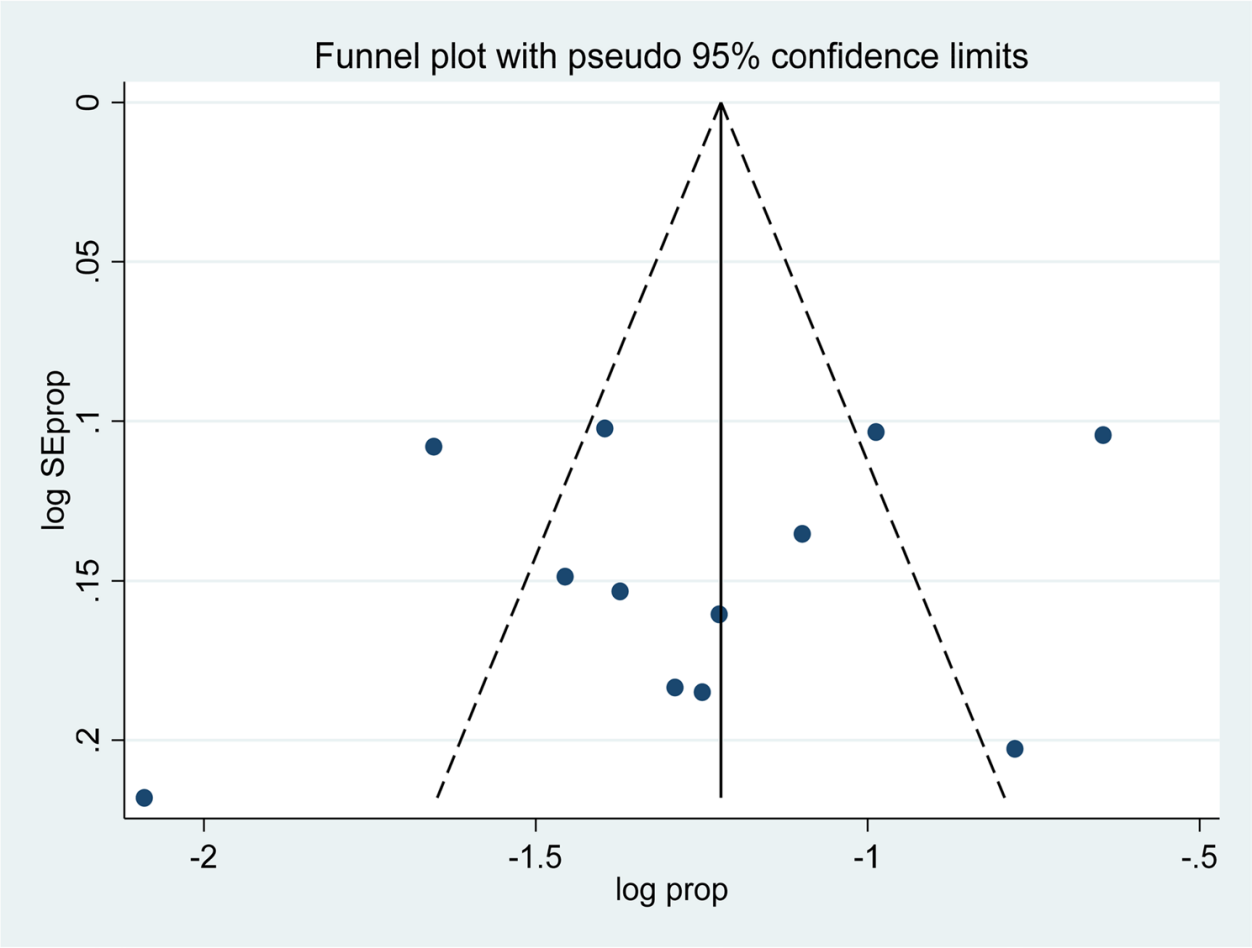
The funnel plot shows symmetry, suggesting no significant publication bias in the studies reporting the prevalence of hypertension among bank workers in Africa, 2024

Among 12 studies in the random effects model, the overall pooled prevalence of hypertension among bank workers in Africa was 29.75 (95% CI = 23.37, 36.12) with a significant heterogeneity observed among studies (I^2^ = 94.4, P-value = 0.000) (Fig. 2). Regarding studies weight, the highest weight among studies observed from the studies conducted by Fikadu G et al. [33]. The symmetry of the funnel plot (See Fig. 3) and Egger’s test results (P-value = 0.438) (See Table 4) confirmed the absence of publication bias in the included studies.

**Table 4:** Egger’s test for small-study effects for the studies of hypertension prevalence among bank workers in Africa, 2024

Number of studies = 12 Root MSE = 2.733					
Standard effect	Coefficient	Standard error	t	P-value	95%CI
Slope	− 0.8933459	0.4194302	−2.13	0.059	−1.827895, 0.0412027
Bias	−2.498973	3.092088	−0.81	***0.438***	−9.388574, 4.390628

### Heterogeneity of the studies

Significant heterogeneity observed from random effects model pooled estimate. To handle this heterogeneity, sensitivity analysis and subgroup analysis were performed.

### Sensitivity analysis

**Fig. 4:**
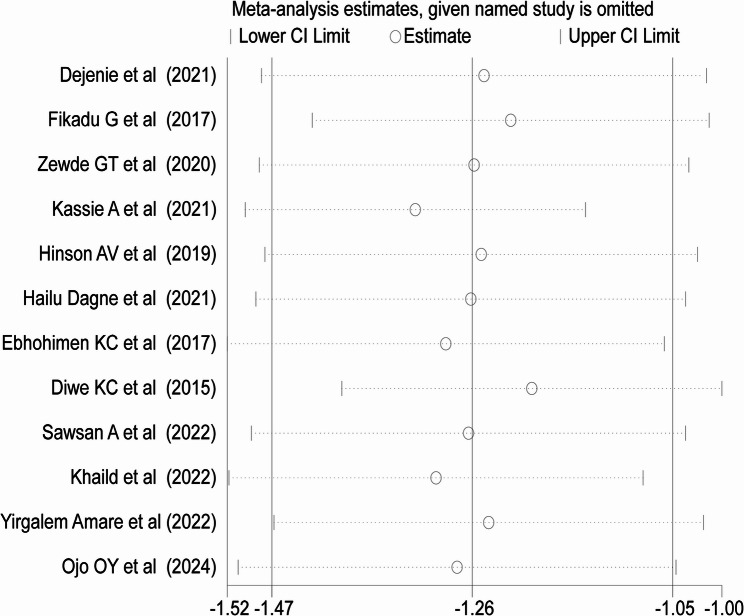
Sensitivity analysis plot for the pooled prevalence of hypertension among bank workers in Africa, 2024

Sensitivity analysis was performed to evaluate the effect of individual studies on the pooled estimate. When individual study was omitted, the pooled prevalence obtained was within the 95% CI of the overall pooled prevalence. This confirms the absence of single study impact on the overall pooled effect size. Therefore, from the random effects model, there were no studies that excessively influence the overall pooled estimate of hypertension (Fig. 4).

### Sub-group analysis

Subgroup analysis conducted among sex, sub-region, country, and publication year.

### Hypertension by sex

**Fig. 5:**
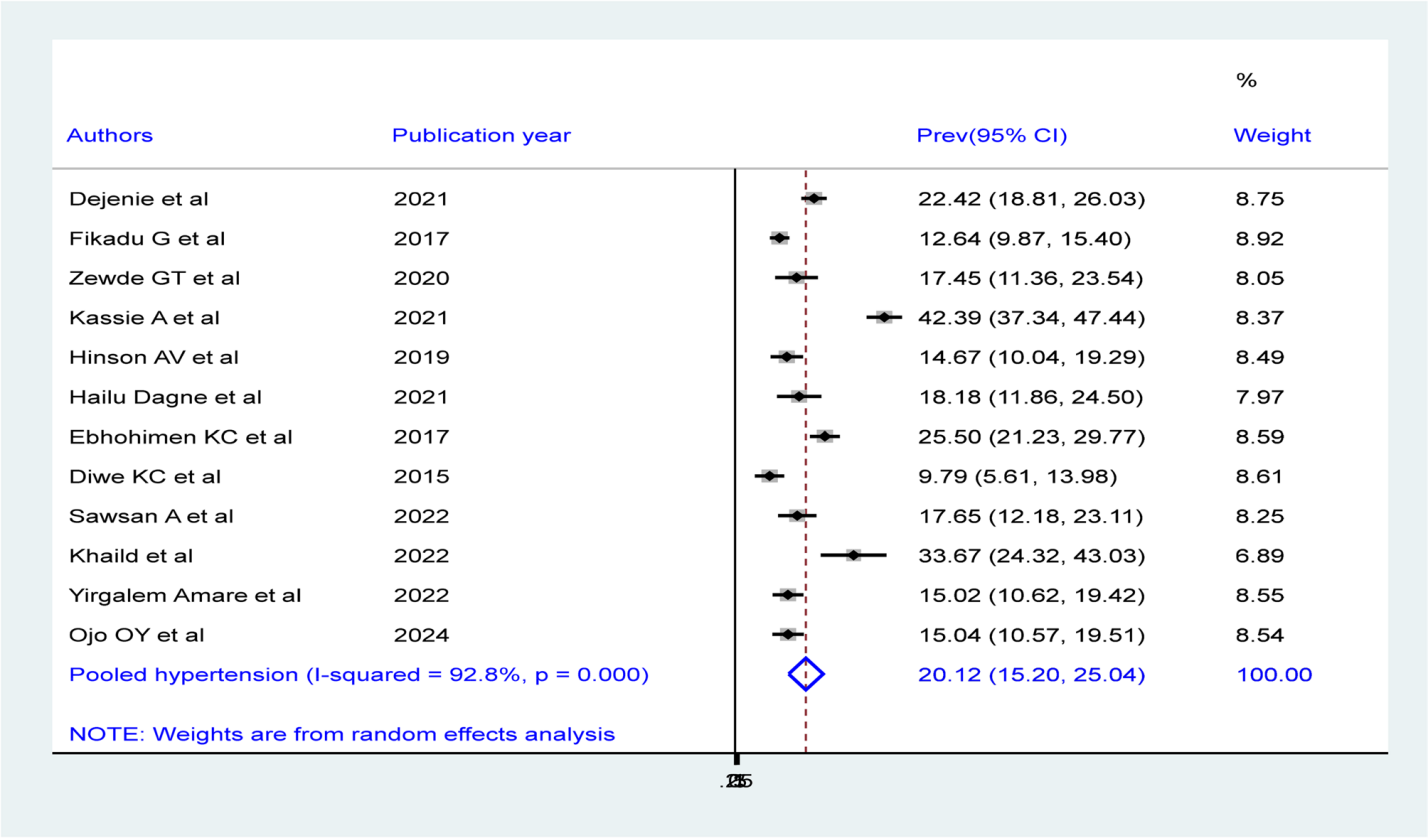
Forest plot showing the pooled prevalence of hypertension among male bank workers in Africa, derived from a random-effects model analysis (2024)

**Fig. 6:**
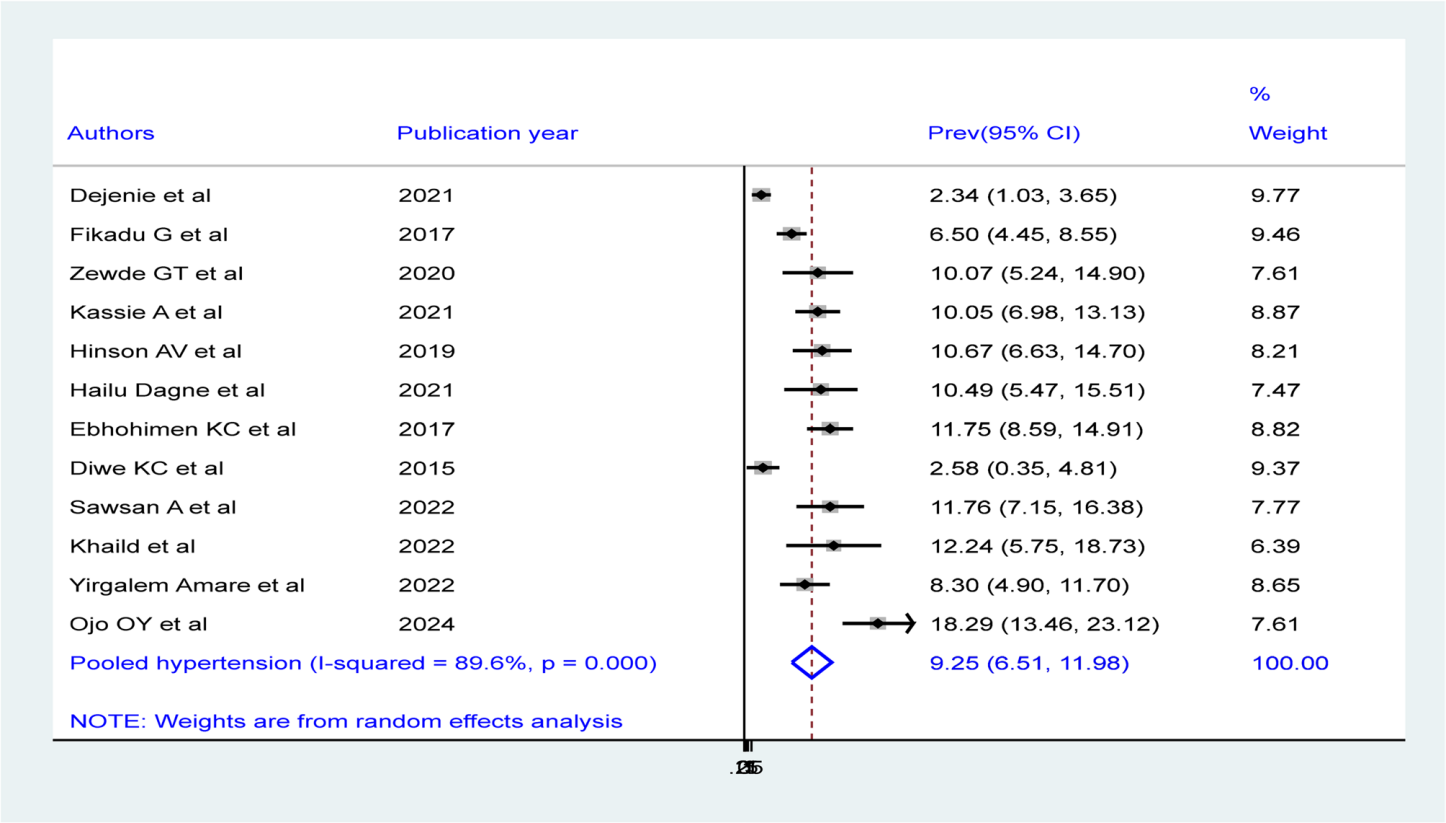
Forest plot showing the pooled prevalence of hypertension among female bank workers in Africa, derived from a random-effects model analysis, 2024

Among 12 studies in the random effects model, the pooled prevalence of hypertension among males of bank workers were 20.12% (95%CI: 15.2, 25.04) (Fig. 5) with statistically significant heterogeneity (I^2^ = 92.8%, P-value = 0.00). Besides, the overall pooled prevalence of hypertension among females of bank workers were 9.25% (95%CI: 6.51, 11.98) with statistically significant heterogeneity (I^2^ = 89.6%, P-value = 0.00) (Fig. 6).

### Hypertension by sub-region

**Fig. 7:**
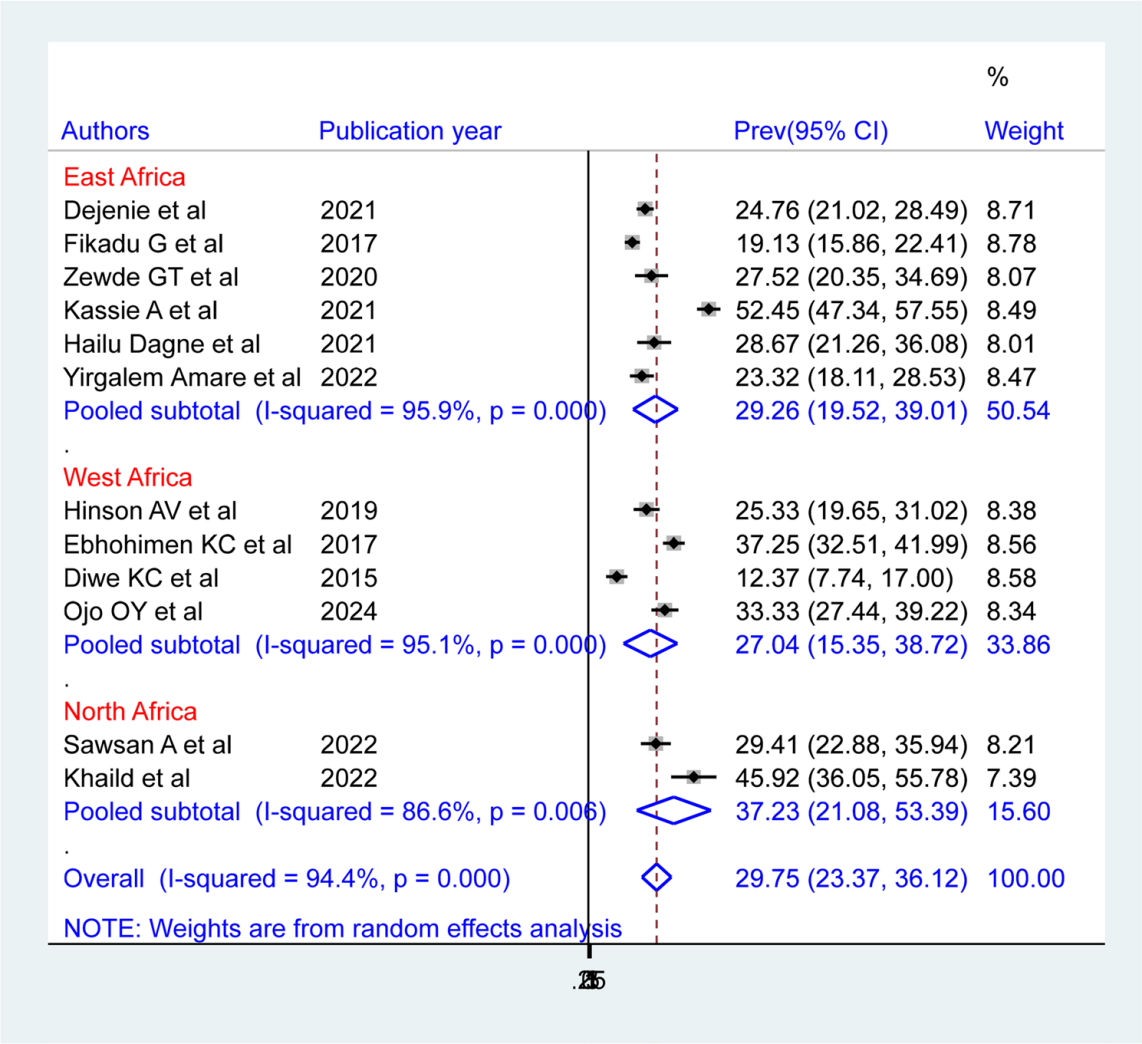
Forest plot showing the pooled prevalence of hypertension among bank workers in Africa, by subregion, based on a random-effects model analysis, 2024

The sub-regional subgroup analysis revealed that North Africa had the highest pooled prevalence of hypertension among African bank workers (37.23% (95% CI: 21.08, 53.39)), followed by East Africa (29.26% (95% CI: 19.52, 39.01)) and West Africa (27.04% (95% CI: 15.35, 38.72)). The East African sub region displayed a high degree of heterogeneity (I^2^ = 95.9%, P-value = 0.00), followed by West African sub region (I^2^ = 95.1%, P-value = 0.00) and North African sub region (I^2^ = 86.6%, P-value = 0.00)(Fig. 7).

### Hypertension by country

**Fig. 8:**
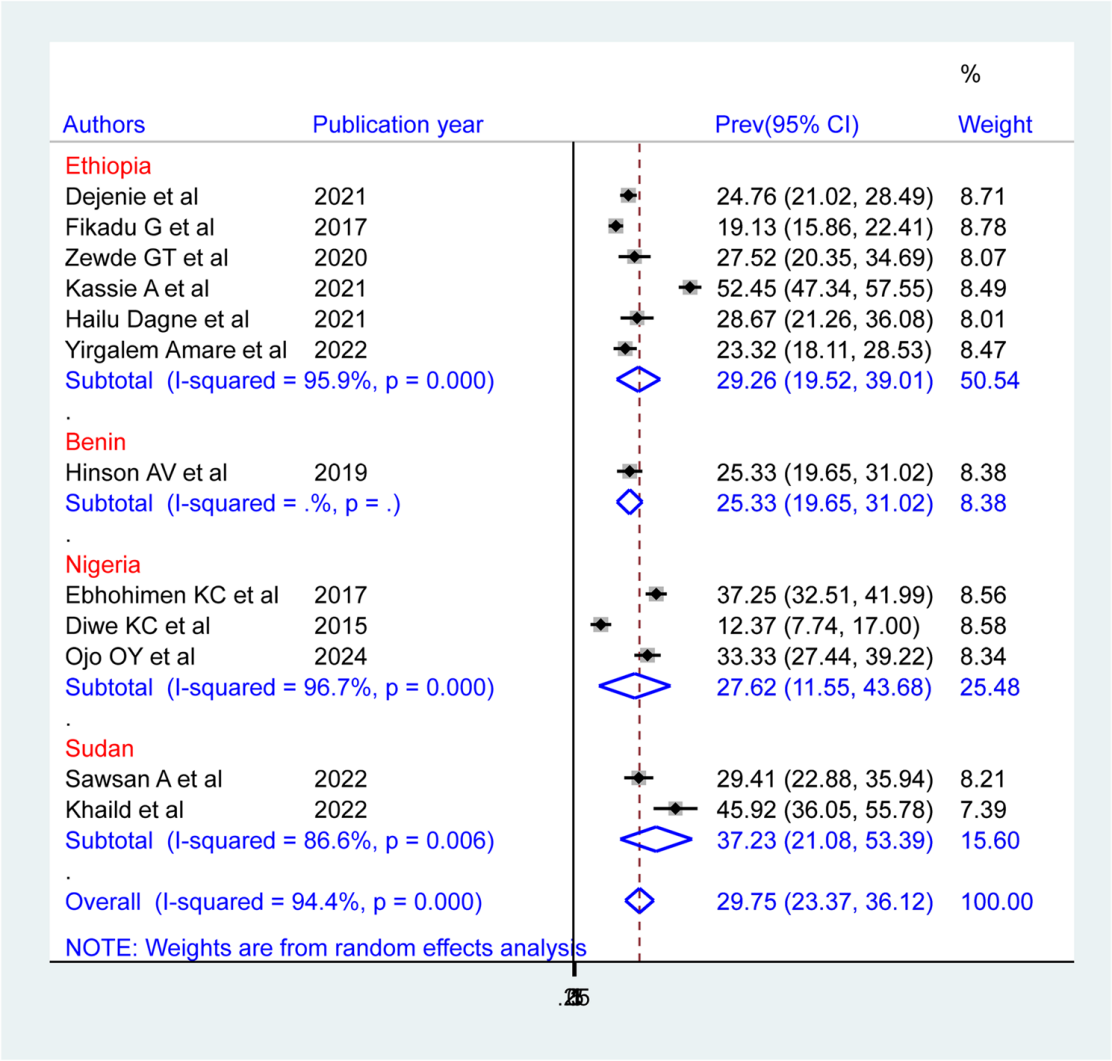
Forest plot showing the pooled prevalence of hypertension among bank workers in Africa, by country, based on a random-effects model analysis, 2024

The subgroup analysis by country indicated that the pooled prevalence of hypertension among bank workers in Africa was highest in Sudan (37.23% (95% CI: 21.08, 53.39)), followed by Ethiopia (29.26% (95% CI: 19.52, 39.01)) and Nigeria (27.62% (95% CI: 11.55, 43.68)). The high degree of heterogeneity seen in Nigeria (I^2^ = 96.7%, P-value = 0.00), followed by Ethiopia (I^2^ = 95.9%, P-value = 0.00) and Sudan (I^2^ = 86.6%, P-value = 0.00) (Fig. 8).

### Hypertension by publication year

**Fig. 9:**
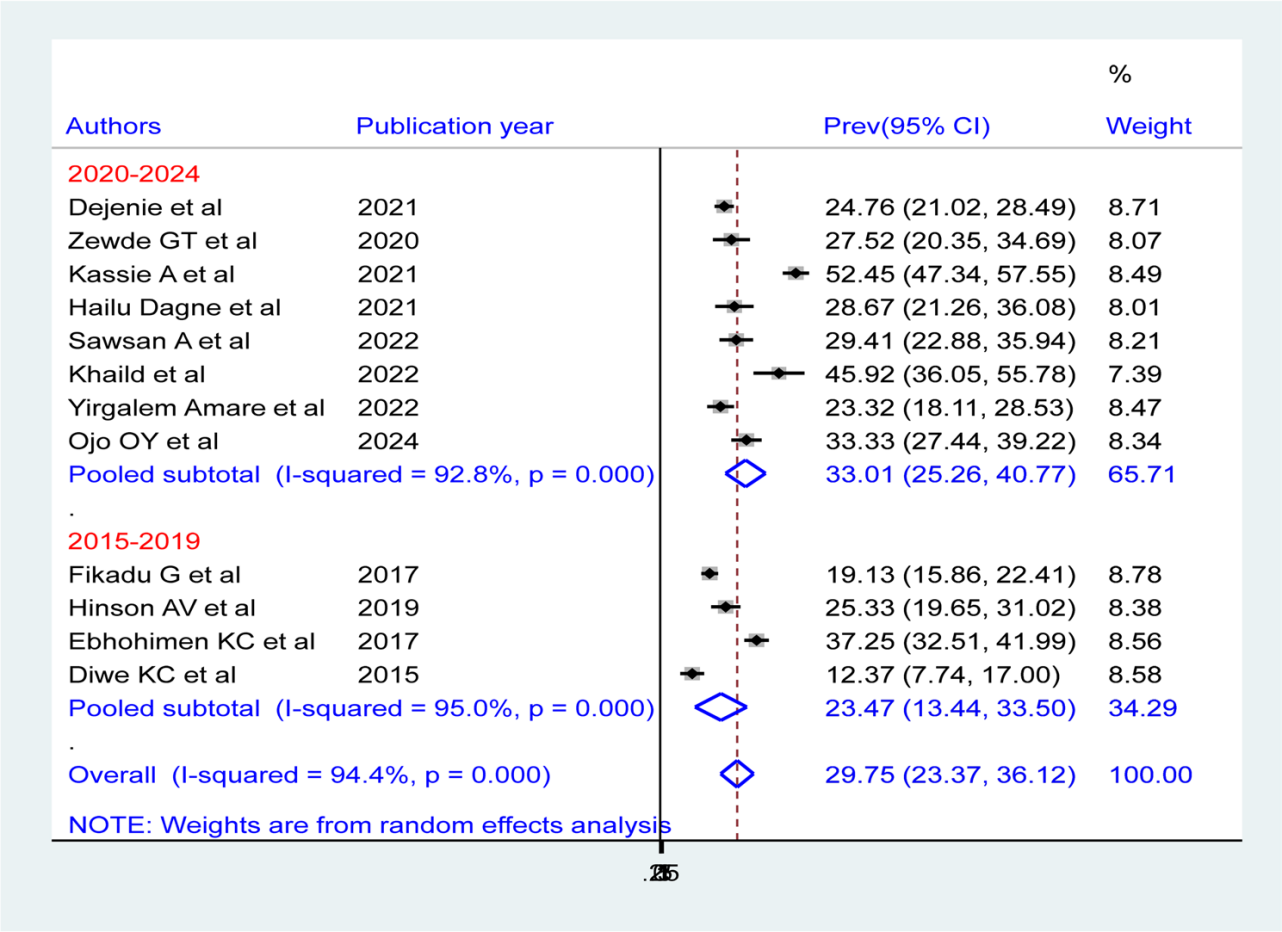
Forest plot showing the pooled prevalence of hypertension among bank workers in Africa, by year of publication, based on a random-effects model analysis, 2024

According to the results of the sub-group analysis by publication year, the pooled prevalence of hypertension was highest (33.01%, 95% CI: 25.26, 40.77)) in 2020–2024 and lowest (23.47%, 95% CI: 13.44, 33.50)) in 2015–2019. Significant heterogeneity was observed in both time periods (2015–2019: I²=95%, *p* < 0.00; 2020–2022: I²=92.8%, *p* < 0.00) (Fig. 9).

### Factors associated with hypertension

In this systematic review and meta-analysis, we identified a variety of factors associated with hypertension among work workers. As summarized in table, poor knowledge, having family history of hypertension, living in sedentary life style, being overweight/obese body mass index and being inactive physical activity are the predictors for hypertension among bank workers, whereas sex was not associated for hypertension among bank workers (Table 5).

**Table 5:** Summary of the pooled effects of factors associated with hypertension among bank workers in Africa, 2024

Variables		OR (95% CI)	Heterogeneity (I^2^, *P*-value)	Total studies	Sample size
Sex	Female	1			
	Male	2.30 [0.95, 5.55]	I² = 79%, *P* = 0.028	2	320
Knowledge	Good	1			
	Poor	**3.55 [2.45**, 5.14] *	I² = 0%, *P* = 0.87	2	738
Family history of hypertension	Yes	**4.57 [1.88**,** 11.12]**	I² = 75%, *P* = 0.04	2	618
	No	**1**			
Sedentary life style	No	1			
	Yes	**2.84 [1.58**, 5.12] *	I² = 0%, *P* = 1.00	2	298
Body mass index	Normal/underweight	1			
	Overweight/obesity	**4.01 [2.94**, 5.47] *	I² = 28%, *P* = 0.24	5	1369
Physical activity	Active	1			
	Inactive	**3.81 [2.70**, 5.38] *	I² = 0%, *P* = 0.66	3	1127

.

### Association between knowledge and hypertension

**Fig. 10:**
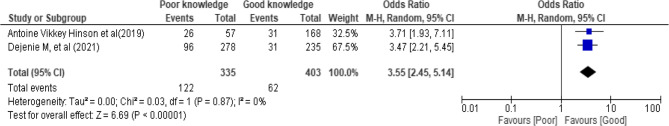
Forest plot showing the association between knowledge and hypertension among bank workers in Africa from pooled estimate of effect size analysis, 2024

A total of two [17, 39] studies included to estimate the association between knowledge and hypertension. Overall, 738 subjects included to analyze the association of knowledge with hypertension. The results of the test statistics indicate that there is no significant heterogeneity was observed between studies (I^2^ = 0%, P-value = 0.87). From the random effects model estimate, the likelihood of developing hypertension among poor knowledge individuals was 3.55 times higher than the individuals with good knowledge towards hypertension (OR = 3.55, 95% CI: 2.45,5.14) (Fig. 10).

### Association between family history of hypertension and hypertension

**Fig. 11:**
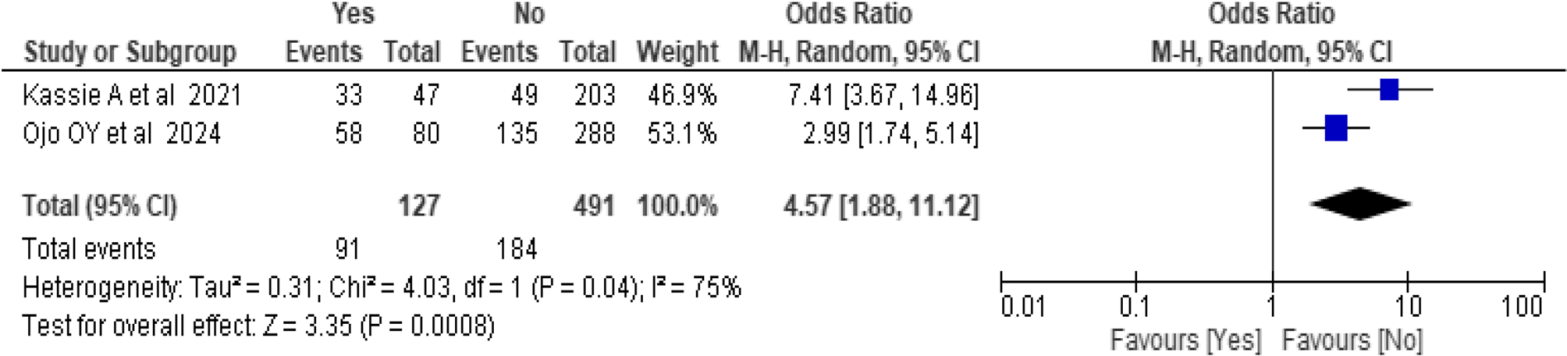
Forest plot showing the association between family history of hypertension and hypertension among bank workers in Africa from pooled estimate of effect size analysis, 2024.

A total of two [36, 38] studies included to estimate the association between familial history of hypertension and hypertension. Overall, 618 subjects included to analyze the association of family history of hypertension with hypertension. The results of the test statistics indicate that there is high significant heterogeneity was observed between studies (I^2^ = 75%, P-value = 0.04). Based on the random-effects model, individuals with a family history of hypertension had 4.57 times higher odds of developing hypertension compared to those without a familial history (OR = 4.57, 95% CI: 1.88–11.12) (Fig. 11).

### Association between physical activity and hypertension

**Fig. 12:**
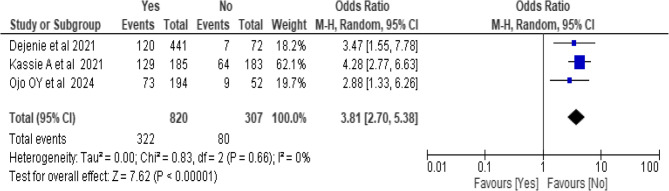
Forest plot showing the association between physical activity and hypertension among bank workers in Africa from pooled estimate of effect size analysis, 2024

A total of two [17, 36, 38] studies included to estimate the association between physical activities and hypertension. A total of 1127 subjects included to analyze the association of physical inactivity with hypertension. The results of the test statistics indicate that there is no significant heterogeneity was observed between studies (I^2^ = 0%, P-value = 0.66). From the random effects model estimate, the odds of developing hypertension among physically inactive individuals were 3.81 compared to the individuals with physically active (OR = 3.81, 95% CI: 2.70, 5.38) (Fig. 12).

### Association of sedentary life style with hypertension

**Fig. 13:**
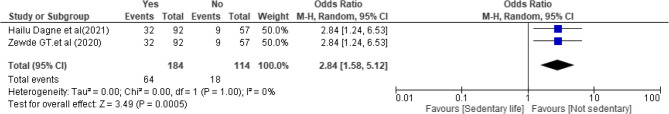
Forest plot showing the association between sedentary life style and hypertension among bank workers in Africa from pooled estimate of effect size analysis, 2024

A total of two [31, 34] studies included to estimate the association between sedentary life style and hypertension. A total of 298 subjects included to analyze the association of sedentary life style with hypertension. The results of the test statistics indicate that there is no significant heterogeneity was observed between studies (I^2^ = 0%, P-value = 1.00). Based on the random effects model, individuals with a sedentary lifestyle had 2.84 times higher odds of developing hypertension compared to those without a sedentary lifestyle (OR = 2.84, 95% CI: 1.58–5.12) (Fig. 13).

### Association of overweight/obesity with hypertension

**Fig. 14:**
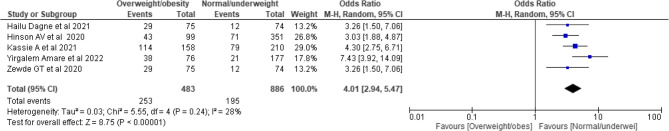
Forest plot showing the association between body mass index and hypertension among bank workers in Africa from pooled estimate of effect size analysis, 2024

A total of five [29, 31, 34, 38, 39] studies included to estimate the association between body mass index and hypertension. A total of 1369 subjects included to analyze the association of overweight/obesity with hypertension. The results of the test statistics indicate that there is moderate heterogeneity was observed between studies (I^2^ = 28%, P-value = 0.24). From the random effects model estimate, the odds of developing hypertension among overweight/obesity individuals were 4.01 compared to the individuals with normal/underweight (OR = 4.01, 95% CI: 2.94, 5.47) (Fig. 14).

## Discussion

Hypertension is emerging as a significant public health concern in Africa, with nearly one in three bank workers affected, according to this review. The prevalence varies across regions, with North Africa reporting the highest rates and West Africa the lowest. Gender disparities were evident, with males more affected than females, while country-specific review highlighted Sudan as having the highest prevalence and Nigeria the lowest. Key risk factors identified include poor knowledge about hypertension, a family history of the hypertension, sedentary lifestyles, overweight or obesity, and low physical activity levels.

This systematic review and meta-analysis revealed that the pooled prevalence of hypertension among bank workers in Africa is 29.75 (95% CI = 23.37, 36.12), which was consistent with a study conducted in Central Province of Sri Lanka 31.7% [52], Bangladesh 24.4% [53], Western India, 26% [54], and in Finland 24% [55]. However, the finding of this meta-analysis was lower than the previous studies conducted in Ghana 38% [56], Bangladeshi 59.9% [57] and Ireland 41.2% [58]. The prevalence of hypertension in this review was higher than a study conducted in Byblos, Lebanon 16.9% [59], in India 10.1% [60], in Iran 4.8% [61]. This discrepancy may be due to the sociodemographic differences, a sedentary life, varying level of stress, and different guidelines to define hypertension.

There were several possible reasons for the high degree of heterogeneity (I² = 94.4%) found in this investigation. It may have resulted from variations in the prevalence among study participants’ sexes, as well as from differences in country, year of publication, and sub-regions of Africa. We therefore considered post-hoc subgroup analyses based on various characteristics, including country, year of publication, sex of study participants, and sub-regions of Africa. Based on subgroup analysis by African sub-regions, the random effects model showed that North Africa had the highest pooled prevalence of hypertension among bank workers (37.23%: 95% CI: 21.08, 53.39), while West Africa had the lowest (27.04%: 95% CI: 15.35, 38.72).

Subgroup analysis by country indicated that Nigeria had the lowest pooled hypertension prevalence (27.62%; 95% CI: 11.55–43.68), while Sudan had the highest (37.23%; 95% CI: 21.08–53.39). These differences may be attributed to variations in study populations, hypertension measurement protocols, sedentary behavior, the number of available studies, sample sizes, and genetic predispositions.

Subgroup analysis by sex revealed a higher prevalence of hypertension in males (20.12%; 95% CI: 15.20–25.04) compared to females (9.25%; 95% CI: 6.51–11.98). Additionally, analysis by publication year indicated an increased pooled hypertension prevalence in 2020– 2024 (33.01%; 95% CI: 25.26–40.77) relative to 2015–2019 (23.47%; 95% CI: 13.44–33.50). While some of the previously mentioned factors may explain this difference, substantial heterogeneity persisted across subgroups.

This systematic review also identified predictors of hypertension. The random effects model pooled estimate revealed that poor knowledge, a family history of hypertension, a sedentary lifestyle, overweight/obese body mass index, and low physical activity levels were significant predictors of hypertension among bank workers in Africa.

The analysis revealed that respondents with poor hypertension knowledge were 3.55 times more likely to have hypertension compared to their well-informed counterparts. This finding aligned with previous research from Cracow [62, 63]. Participants with better hypertension knowledge likely maintained healthier lifestyles and demonstrated more proactive health-seeking behaviors. The observed disparity potentially stemmed from variations in health literacy levels across study populations. Furthermore, the protective association among knowledgeable participants may reflect their enhanced capacity for health-related decision-making. This advantage potentially enabled them to mitigate exposure to hypertension risk factors, particularly those affecting cardiovascular health.

The review identified physical inactivity as a significant risk factor for hypertension among bank workers. Individual with inactive physical activity were 3.81 times more likely to have hypertension than those performed regular physical exercise. This finding is supported by existing evidence indicating that regular physical activity helps maintain healthy blood pressure levels by improving vascular compliance and reducing arterial stiffness [64].

The review also revealed, sedentary life style had association factors for hypertension among bank workers. Bank workers who had sedentary lifestyle were 2.84 times more likely to have hypertension than those who had no sedentary life. This findings aligned with, study conducted among bank workers in Surratt city of India which showed that individual with sedentary life style had 2 times risked for hypertension than those who had no sedentary life [65]. This shows that sedentary life decreases energy expenditure and increase cholesterol level in blood vessel which directly related with hypertension.

Additionally, this review showed that individuals who were overweight or obese had a higher odd of developing hypertension. The odds of developing hypertension were 4.01 times higher in those with a body mass index was overweight or obese than in the normal population. The results of earlier research done in various nations [67–70] are comparable to this one. While the exact mechanism by which obesity causes hypertension remains unclear, several factors are thought to play a significant role in the pathophysiology of obesity-related hypertension, including the renin-angiotensin system, the amount of intra-abdominal and intravascular fat, sodium retention that increases renal reabsorption, and the sympathetic nervous system activation [70].

Furthermore, this review showed that individuals who had family history of hypertension had a higher odd of developing hypertension. Bank workers with a family history of hypertension had 4.57 times higher odds of developing hypertension compared to those without a familial history. This findings aligned with previous study conducted in India [71], Saudi Arabia [72], Bangladesh [53], Pakistan [73], Ethiopia [74], low and middle income countries [75]. Epidemiological studies also indicated that individuals with a family history of hypertension face increased risk of developing hypertension compared to those without such familial predisposition in low and middle income countries [76]. Furthermore, the Johns Hopkins Precursors Study demonstrated that parental hypertension (both maternal and paternal) showed significant independent associations with elevated blood pressure trajectories and incident hypertension throughout adulthood [77].

## Conclusion and recommendations

In conclusion, hypertension is becoming a major public health problem in Africa. Nearly one over three bank workers in Africa are living with hypertension. The highest prevalence of hypertension was observed in North Africa and the lowest was observed in West Africa, regarding sex highest in male than female, and by country highest in Sudan and the lowest was in Nigeria, as well as by year of publication, highest in 2020– 2024 than in 2015–2019. Poor knowledge, family history of hypertension, living in sedentary life style, being overweight/obese body mass index and being inactive physical activity were statistically significant factors for hypertension among bank workers in Africa.

Based on the findings of this review, we recommend that health planners, policymakers, and the banking community prioritize addressing behavioral risk factors particularly sedentary lifestyles, physical inactivity, and overweight/obesity among bank workers. Given the high prevalence of hypertension in this occupational group, regular health screenings and awareness campaigns are critical. These efforts should emphasize the asymptomatic nature of hypertension and the protective role of physical exercise in reducing body mass index (BMI) and preventing elevated blood pressure. By targeting modifiable behaviors through education and workplace interventions, stakeholders can mitigate the growing burden of hypertension in this high-risk population.

To ensure lasting impact, a multi-level approach is essential. At the institutional level, workplaces should implement routine blood pressure monitoring, introduce ergonomic solutions (e.g., standing desks), and mandate scheduled movement breaks to counteract prolonged sitting. Beyond individual workplaces, policymakers must advocate for national health policies tailored to high-risk sectors like banking, where sedentary work is endemic. Simultaneously, strengthening primary healthcare systems to support early detection and continuous management of hypertension is vital.

### Strengths and limitations

This study follows some strengths and limitations. Our review adds considerable knowledge of the updated prevalence of hypertension among bank workers in Africa which did not investigate before. Almost all included studies use the same definition to declare hypertension. Subgroup analysis was performed to minimize statistical heterogeneity. Multiple factors were also included to identify the significant factors for hypertension among bank workers.

However, this systematic review and meta-analysis on hypertension prevalence and associated factors among bank workers primarily included cross-sectional studies, which are prone to potential confounding, selection bias, recall bias, social desirability bias, potentially skewing self-reported data and the inability to establish causality. The absence of randomized controlled trials (RCTs) or quasi-experimental designs means that observational studies which inherently lack random assignment cannot establish causality with high certainty.

Although significant variation was observed among the included studies, considerable heterogeneity remained even after subgroup analysis. The review was limited to English-language publications and included only twelve observational studies from four African countries (Ethiopia, Benin, Nigeria, and Sudan). Additionally, access to databases such as Scopus, HINARI, EMBASE, CINAHL, and Web of Science was restricted. Among the included studies, three utilized outdated hypertension diagnostic criteria (≥ 130/90 mmHg) based on the American Heart Association guidelines, rather than the current joint national committee 7 classification threshold (≥ 140/90 mmHg).

## Supplementary Information

Supplementary material 1.

## Data Availability

.All generated or analyzed datasets are presented within the manuscript.

## Abbreviations

AJOL: African J ournals Online
AOR: Adjusted Odd Ratio
BMI: Body Mass Index
CI: Confidence Interval
CINAHL: Cumulative Index to Nursing and Allied Health Literature
CVDs: Cardiovascular Diseases
DALYs: Disability-Adjusted Life-Years
DM: Diabetic Mellitus
GDP: Gross Domestic Product
HTN: Hypertension
ID: Identification
JBI: Joanna Briggs Institute
LMIC: Low- and Middle-Income Countries
MeSH: Medical Subject Headings
METs: Metabolic Equivalents
NCD: Non-Communicable Disease
PASCAR: Pan-African Society of Cardiology
PRISMA-P: Preferred Reporting Items for Systematic Review and Meta-Analysis Protocols
USD: United States Dollar
